# Potential of conventional & bispecific broadly neutralizing antibodies for prevention of HIV-1 subtype A, C & D infections

**DOI:** 10.1371/journal.ppat.1006860

**Published:** 2018-03-05

**Authors:** Kshitij Wagh, Michael S. Seaman, Marshall Zingg, Tomas Fitzsimons, Dan H. Barouch, Dennis R. Burton, Mark Connors, David D. Ho, John R. Mascola, Michel C. Nussenzweig, Jeffrey Ravetch, Rajeev Gautam, Malcolm A. Martin, David C. Montefiori, Bette Korber

**Affiliations:** 1 Theoretical Biology & Biophysics, Los Alamos National Laboratory, Los Alamos, United States of America; 2 New Mexico Consortium, Los Alamos, United States of America; 3 Center for Virology & Vaccine Research, Beth Israel Deaconness Medical Center, Boston, United States of America; 4 Department of Immunology and Microbiology, The Scripps Research Institute, La Jolla, United States of America; 5 Laboratory of Immunoregulation, National Institute of Allergy and Infectious Diseases, National Institutes of Health, Bethesda,United States of America; 6 Aaron Diamond AIDS Research Center, The Rockefeller University, New York, United States of America; 7 Vaccine Research Center, National Institute of Allergy and Infectious Diseases, National Insitutes of Health, Bethesda, United States of America; 8 Laboratory of Molecular Immunology, The Rockefeller University, New York, United States of America; 9 Laboratory of Molecular Genetics and Immunology, The Rockefeller University, New York, United States of America; 10 Laboratory of Molecular Microbiology, National Institute of Allergy and Infectious Diseases, National Institutes of Health, Bethesda, United States of America; 11 Department of Surgery, Duke University Medical Center, Durham, United States of America; University of Zurich, SWITZERLAND

## Abstract

There is great interest in passive transfer of broadly neutralizing antibodies (bnAbs) and engineered bispecific antibodies (Abs) for prevention of HIV-1 infections due to their *in vitro* neutralization breadth and potency against global isolates and long *in vivo* half-lives. We compared the potential of eight bnAbs and two bispecific Abs currently under clinical development, and their 2 Ab combinations, to prevent infection by dominant HIV-1 subtypes in sub-Saharan Africa. Using *in vitro* neutralization data for Abs against 25 subtype A, 100 C, and 20 D pseudoviruses, we modeled neutralization by single Abs and 2 Ab combinations assuming realistic target concentrations of 10μg/ml total for bnAbs and combinations, and 5μg/ml for bispecifics. We used IC_80_ breadth-potency, completeness of neutralization, and simultaneous coverage by both Abs in the combination as metrics to characterize prevention potential. Additionally, we predicted *in vivo* protection by Abs and combinations by modeling protection as a function of *in vitro* neutralization based on data from a macaque simian-human immunodeficiency virus (SHIV) challenge study. Our model suggests that nearly complete neutralization of a given virus is needed for *in vivo* protection (~98% neutralization for 50% relative protection). Using the above metrics, we found that bnAb combinations should outperform single bnAbs, as expected; however, different combinations are optimal for different subtypes. Remarkably, a single bispecific 10E8-iMAb, which targets HIV Env and host-cell CD4, outperformed all combinations of two conventional bnAbs, with 95–97% predicted relative protection across subtypes. Combinations that included 10E8-iMAb substantially improved protection over use of 10E8-iMAb alone. Our results highlight the promise of 10E8-iMAb and its combinations to prevent HIV-1 infections in sub-Saharan Africa.

## Author summary

In the absence of effective vaccines, the use of passive transfer of conventional and engineered antibodies to prevent HIV-1 infection is being considered. This approach is promising because of broad efficacy and long *in vivo* lifetimes of antibodies. We analyzed the potential of leading antibody candidates, and combinations of two antibodies, to prevent HIV-1 infections in sub-Saharan Africa, the hardest-hit region in the world. We used *in vitro* antibody neutralization data to predict neutralization metrics that might be relevant for *in vivo* success, and modeled antibody-based *in vivo* protection as a function of *in vitro* neutralization using data from a macaque study. By systematic comparison, we found, as expected, that combinations of two conventional antibodies significantly outperformed individual conventional antibodies, even with same total concentration. However, different antibody combinations were optimal for the different HIV-1 subtypes analyzed. The engineered bispecific 10E8-iMAb, which targets epitopes on HIV Env and host-cell CD4, was predicted to reduce infection probability by 20–30 fold, and outperformed all individual antibodies and combinations of two conventional antibodies. This performance was further improved by combining 10E8-iMAb with other antibodies. Thus, our results suggest that passive transfer of current antibody candidates, especially 10E8-iMAb and its combinations, might be successful in prevention of HIV-1 infections in sub-Saharan Africa.

## Introduction

The World Health Organization estimated that in 2015, approximately two-thirds of the 2 million new HIV-1 infections globally, were in sub-Saharan Africa. Since HIV-1 infection cannot be cured, effective vaccines or other prevention measures are needed to mitigate the impact of HIV/AIDS on global health. Successful antibody (Ab)-based vaccines prevent infection, and T-cell-based vaccines enhance control of infection, but the development of such vaccines has proven challenging [1]. Pre-exposure prophylaxis (PrEP) with reverse transcriptase inhibitors is effective in prevention of HIV-1 infections, and is in current use [2]. PrEP efficacy, however, depends on adherence, which is challenging given that four or more doses a week are required, and associated costs and toxicity [2, 3]. Thus, alternative approaches to PrEP using broadly neutralizing antibodies (bnAbs) or long acting antiretroviral formulations are being explored [4].

Many bnAbs isolated from chronically infected individuals can potently neutralize a substantial fraction of diverse global panels of HIV-1 pseudoviruses *in vitro*. Their characterization has provided insights for vaccine design [5–9], enabling progress in strategies for eliciting bnAb responses [10, 11]. The best bnAbs are also promising candidates for passive transfer to prevent HIV-1 infections, a more readily achievable goal [4, 12].

Several preclinical studies have shown efficacy for prevention of HIV-1 infections following passive transfer of bnAbs [13–21]. In a recent repeated low-dose simian-human immunodeficiency virus (SHIV) challenge study in rhesus macaques using SHIV_AD8-EO_ [21], a single bnAb infusion delayed infection by weekly SHIV challenges to medians of 8–14 weeks, depending on the bnAb, compared to median of 3 weeks for infection of control animals. This underscores a main advantage of bnAbs over most small-molecule drugs–the long *in vivo* half-lives of bnAbs can result in prolonged protection by a single dose. Antibodies can be engineered to extend *in vivo* half-life even further [22, 23]. Other advantages include Fc-mediated effector functions [24, 25], reduced side effects, and the availability of alternative approaches in situations of emerging drug resistance. Based on such encouraging data, several promising bnAbs are being clinically developed, and have either begun (PGT121 in clinical trial NCT02960581 (ClinicalTrials.gov identifier) and VRC07-523LS in NCT03015181), or completed preliminary human testing (VRC01, 3BNC117 and 10–1074) [26–28]. The first phase 2b efficacy trials using the bnAb VRC01 are underway in three continents (NCT0271665, NCT02568215).

**Fig 1:**
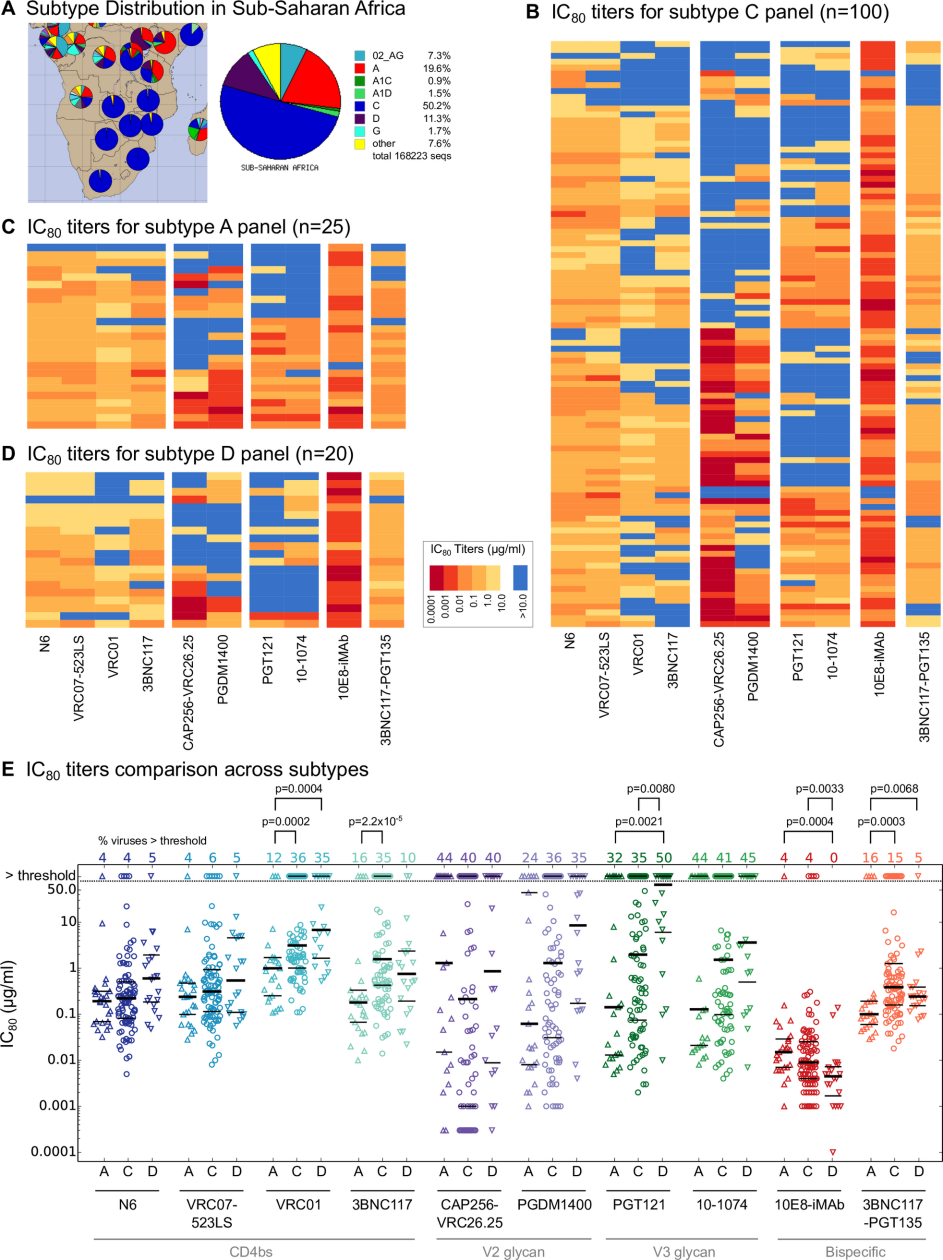
***In vitro* neutralization data for individual antibodies against A, C & D subtype pseudovirus panels.** (A) Distribution of HIV-1 subtypes in sub-Saharan Africa using the “Geography Search Interface” on the Los Alamos HIV Database. (B-D) Experimental IC_80_ titers for antibodies against subtype C, A and D panels, respectively. Viruses are represented on rows and antibodies on columns. Red-yellow shades indicate more-less potent neutralization, and blue cells indicate IC_80_ titers above experimental threshold or 10μg/ml. (E) Comparison of IC_80_ distributions for each antibody across subtypes. The percent of viruses in each subtype with IC_80_ above experimental threshold for each antibody are indicated in the figure. The IC_80_ distributions between subtypes were compared for each antibody using Wilcoxon rank sum test and comparisons with p < 0.01 are indicated.

The potency of particular bnAbs against different pseudoviruses tested in global panels is highly variable (Fig 1); some Envs for any given bnAb will be completely resistant or have less potent IC_80_ titers [29, 30]. Patterns of Env sensitivity are similar for bnAbs targeting similar epitopes, but differ across epitope classes [30]. Thus, a natural solution to the problem of limited breadth/potency is to combine bnAbs targeting different epitopes [29]. Neutralization for bnAb combinations *in vitro* can be very accurately modeled using individual bnAb data, suggesting that bnAbs targeting different epitopes act independently when used in combination [30]. Other solutions include engineering artificial bispecific antibodies with two Fab arms derived from different bnAbs [31–33], or bispecific antibodies with one arm targeting the HIV receptor or co-receptor on host cells, and the other targeting HIV-1 Env [34–36]. A different approach involves arms derived from the CD4 receptor, with the Ab base including a CCR5 co-receptor mimetic peptide [37]. All these approaches can increase neutralization breadth and potency against diverse viruses, and several are under clinical development.

As promising bnAb and bispecific candidates are developed, it will be important to assess their potential for *in vivo* prevention, and to compare *in vivo* performance to *in vitro* measures of neutralization. This will help inform choices regarding candidates Abs for subsequent advancement in the clinical testing pipeline. For successful clinical outcomes, Abs or Ab combinations will need to be effective against diverse circulating strains and the diversity in viral quasispecies that accumulates in each chronically infected donor. Furthermore, genetically identical virus samples can have Ab resistant subpopulations [30, 38], due to phenotypic heterogeneity in glycosylation profiles [39] and protein conformations [40, 41]. Economic factors must also be considered, as Ab manufacturing costs may be higher than for small molecule drugs.

Here, we analyzed the potential of several leading conventional and bispecific Ab candidates to prevent HIV-1 infections in sub-Saharan Africa. We obtained *in vitro* neutralization data for leading Ab candidates against virus panels of subtypes A, C & D, the dominant subtypes in this region, and used our modeling approach to predict neutralization by all Abs and 2 Ab combinations [30]. We compared the *in vitro* performance of Abs and Ab combinations using realistic *in vivo* target concentrations and previously developed metrics [30] that measure breadth-potency of neutralization, and efficacy against within-host viral diversity and viral phenotypic heterogeneity. Finally, we modeled Ab-mediated *in vivo* protection as a function of *in vitro* neutralization using data from a macaque SHIV challenge study, and used this to predict the relative protection afforded by Abs and Ab combinations in this study.

## Results

### *In vitro* neutralization data for subtypes A, C & D

We collected *in vitro* neutralization data (Methods) for leading candidate bnAbs and bispecifics for PrEP: CD4 binding site (CD4bs) bnAbs 3BNC117 [42], N6 [43], VRC01 [44] and VRC07-523LS [23]; V2 glycan (V2g) apex bnAbs CAP256-VRC26.25 [45] and PGDM1400 [46]; V3 glycan (V3g) bnAbs 10–1074 [47] and PGT121 [48]; and bispecific antibodies 10E8_v2.0_-iMAb (10E8-iMAb for brevity) [36], which targets membrane proximal external region (MPER) on Env and host-cell CD4, and 3BNC117-PGT135 [32], which targets CD4 binding site and V3 glycan epitopes on Env.

We studied the efficacy of the above antibodies against subtype A, C and D viruses, which make up 81.1% of the Los Alamos HIV database sequences from sub-Saharan Africa (Fig 1A). The subtype C panel is a 100 pseudovirus subset of a previous panel of 200 early/acute viruses from southern Africa, designed to preserve the breadth-potency profiles of bnAbs as seen for the larger panel [49, 50]. Subtype A panel includes 25 pseudoviruses, subtype D 20, cloned from chronically infected individuals from five sub-Saharan Africa countries each spanning years 1992–2008 and 1993–2008, respectively (S1 Table). 22 out of 25 subtype A pseudoviruses (including 5 transmitted-founder viruses) and 11 out of 20 subtype D viruses (with 4 transmitted-founder viruses) were isolated from acute/early infections. Majority of subtype A and D pseudoviruses were cloned using single genome amplification or limiting dilution PCR. IC_80_ titer heatmaps of antibodies are shown in Fig 1B–1D, and IC_50_ and IC_80_ data are reported in S1 and S2 Data.

These data recapitulate previously observed bnAb neutralization profiles, e.g. [29, 30]: V2g and V3g bnAbs can be very potent, but have limited breadth (Fig 1), and CD4bs bnAbs are generally less potent but show higher breadth. Also, V2g and V3g bnAbs tend to have complementary reactivity patterns; Envs that are insensitive to V3g bnAbs are sensitive to V2g bnAbs, and vice versa. Several Abs showed subtype-specific differences in IC_80_ potency (see Fig 1E for levels of statistical support). CD4bs bnAbs VRC01, 3BNC117 and bispecific 3BNC117-PGT135 were significantly more potent against subtype A viruses; PGT121 less potent against subtype D; and 10E8-iMAb was less potent against subtype A.

### Performance of single antibodies

**Fig 2:**
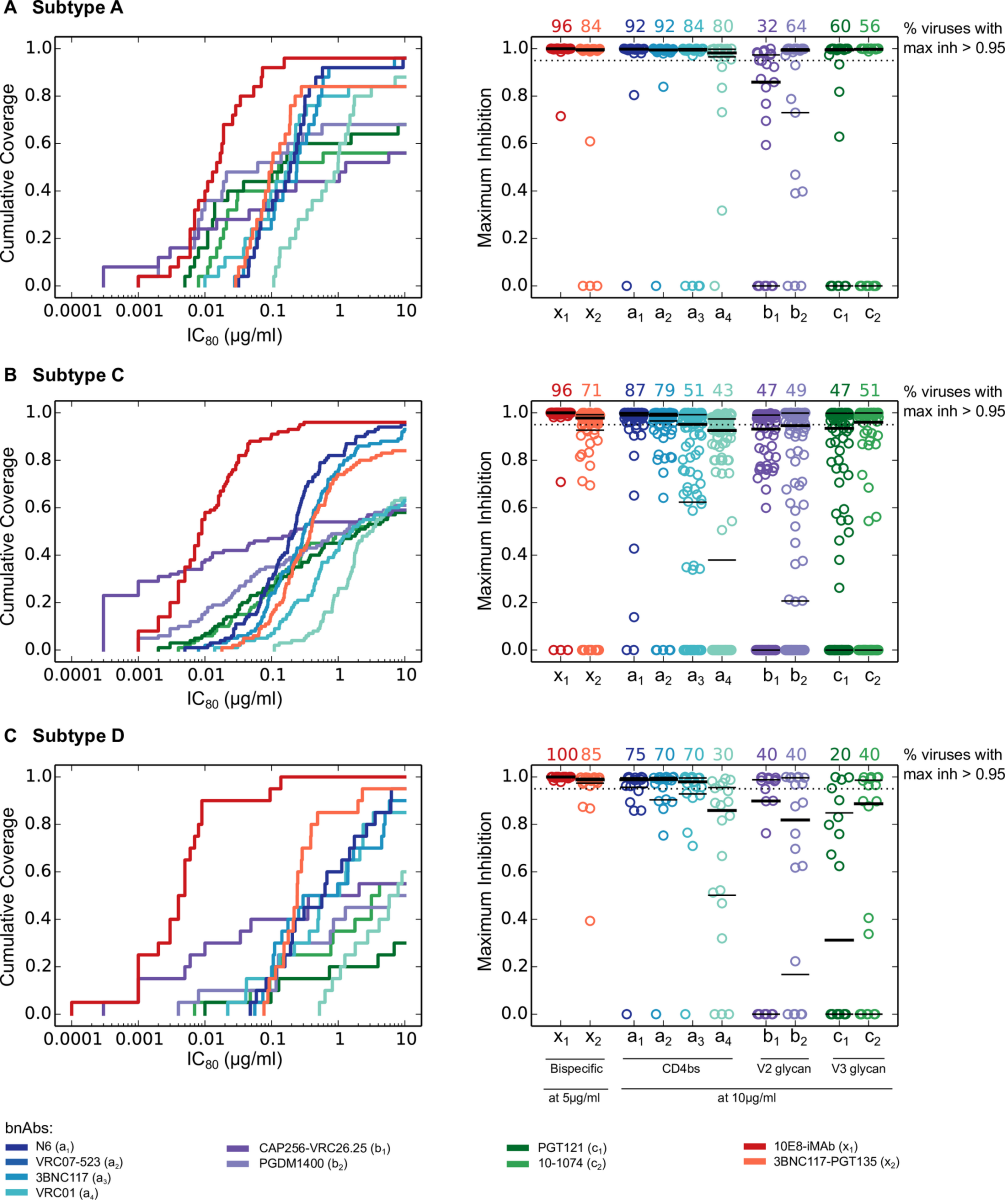
Performance of individual antibodies. (A-C) show the results for subtypes A, C and D, respectively. The left panels show IC_80_ breadth-potency curves for antibodies and the right panels show the fraction maximum inhibition values at 10 μg/ml for conventional antibodies and at 5 μg/ml for bispecifics against viruses from a given subtype virus panel. The numbers on top of right panels show the percent of viruses in the panel that had maximum inhibition > 0.95 for each antibody.

We next characterized the performance of individual Abs using *in vitro* IC_80_ breadth and potency and completeness of neutralization (Fig 2). Some bnAbs incompletely neutralize pseudoviruses even at very high concentrations [30, 38, 51], due to phenotypic heterogeneity in a clonal pseudovirus sample arising from heterogeneity in glycan occupancy and/or processing [39], dynamics of Env trimers [40] and alternate variable loop configurations [41]. We modeled the fraction of pseudovirus neutralized by bnAbs using the Hill curve parametrization of neutralization curves (Methods), which accurately predicts observed neutralization profiles [30]. As exact thresholds for *in vivo* efficacy are not yet well characterized, we used >95% for complete neutralization as before [30]. This threshold is not unreasonably high, as our analysis of a low-dose SHIV challenge study below showed that a few macaques got infected in spite of serum bnAb levels corresponding to 95–99% *in vitro* neutralization of the challenge pseudovirus [21]. In the ongoing Phase 2b VRC01 clinical trials, the minimum VRC01 *in vivo* serum concentrations are predicted to be 5–16 μg/ml [52]. Based on this, we chose the target minimum concentration of 10 μg/ml total for our modeling of Abs, individually or in combinations. We used 5 μg/ml for bispecifics because of their greater potency (Fig 1). Since post-infusion concentrations will be higher than the minimum concentrations we assumed, our results yield conservative estimates of Ab efficacy.

Even with using half the target concentration, 10E8-iMAb had the best neutralization metrics when compared against all other individual Abs for each subtype (Fig 2 and S2 Table). It showed best IC_80_ potency (median IC_80_ of 0.0045–0.015 μg/ml across all subtypes, 4–54 fold more potent than the next most potent Ab, p = 9.4x10-8–0.037 using Wilcoxon rank-sum test) and completely neutralized (>95% neutralization) 96–100% of panel viruses.

For subtypes A and C, the next best performing single Ab was N6, with 95–96% IC_80_ coverage and complete neutralization of 87–92% panel viruses at 10 μg/ml. For subtype D, the bispecific 3BNC117-PGT135 showed the second most potent IC_80_ titers after 10E8-iMAb (median IC_80_ of 0.2425 μg/ml) and completely neutralized 85% of viruses at 5 μg/ml. While V2g bnAbs were very potent against sensitive viruses, they had relatively low IC_80_ breadth (55–59% for CAP256-VRC26.25 and 50–68% for PGDM1400) and lower proportion of completely neutralized viruses (32–47% for CAP256-VRC26.25 and 40–64% for PGDM1400). Similarly, V3g bnAbs, which were slightly less potent than V2g, also show limited IC_80_ breadth and a low proportion of viruses completely neutralized. Among CD4bs bnAbs, N6 was best, VRC07-523LS was nearly comparable followed by 3BNC117 and VRC01 (Fig 2).

To partially mitigate potential sampling biases of our pseudovirus panels (particularly the smaller subtype A and D panels), we performed bootstrap resampling to understand the robustness of our results (Methods). We generated 1,000 bootstrap realizations to match the size of each panel, and characterized the median and 95% confidence intervals (CI) for each of the above metric for each Ab; these results are presented in S3 Table. We found that 10E8-iMAb still showed the best metrics for all subtypes, with few bnAbs showing any metrics that were within the bootstrap 95% CI of the respective metric for 10E8-iMAb (S3 Table).

### Performance of 2 bnAb combinations

Next, we analyzed combinations of 2 conventional bnAbs, since combinations improve performance over single bnAbs [29, 30]. We used the Bliss-Hill model on single bnAb IC_50_ and IC_80_ data to predict combination IC_80_ titers and pseudovirus fraction neutralized for combinations of 2 bnAbs targeting different epitopes (Methods). This approach was shown to accurately predict experimental data [30]. We assumed equal concentrations for both bnAbs, and used a total target concentration of 10 μg/ml for the combination, i.e. 5μg/ml per bnAb. As before, we used IC_80_ breadth-potency and completeness of neutralization as metrics to evaluate performance. We also used coverage with both bnAbs active as a metric for prevention success, assuming a virus is actively neutralized by both bnAbs in a combination if IC_80_ < 5μg/ml for each bnAb individually. The rationale behind this metric is that strains from within-host quasispecies will have a lower chance of resistance to both bnAbs [30].

**Fig 3:**
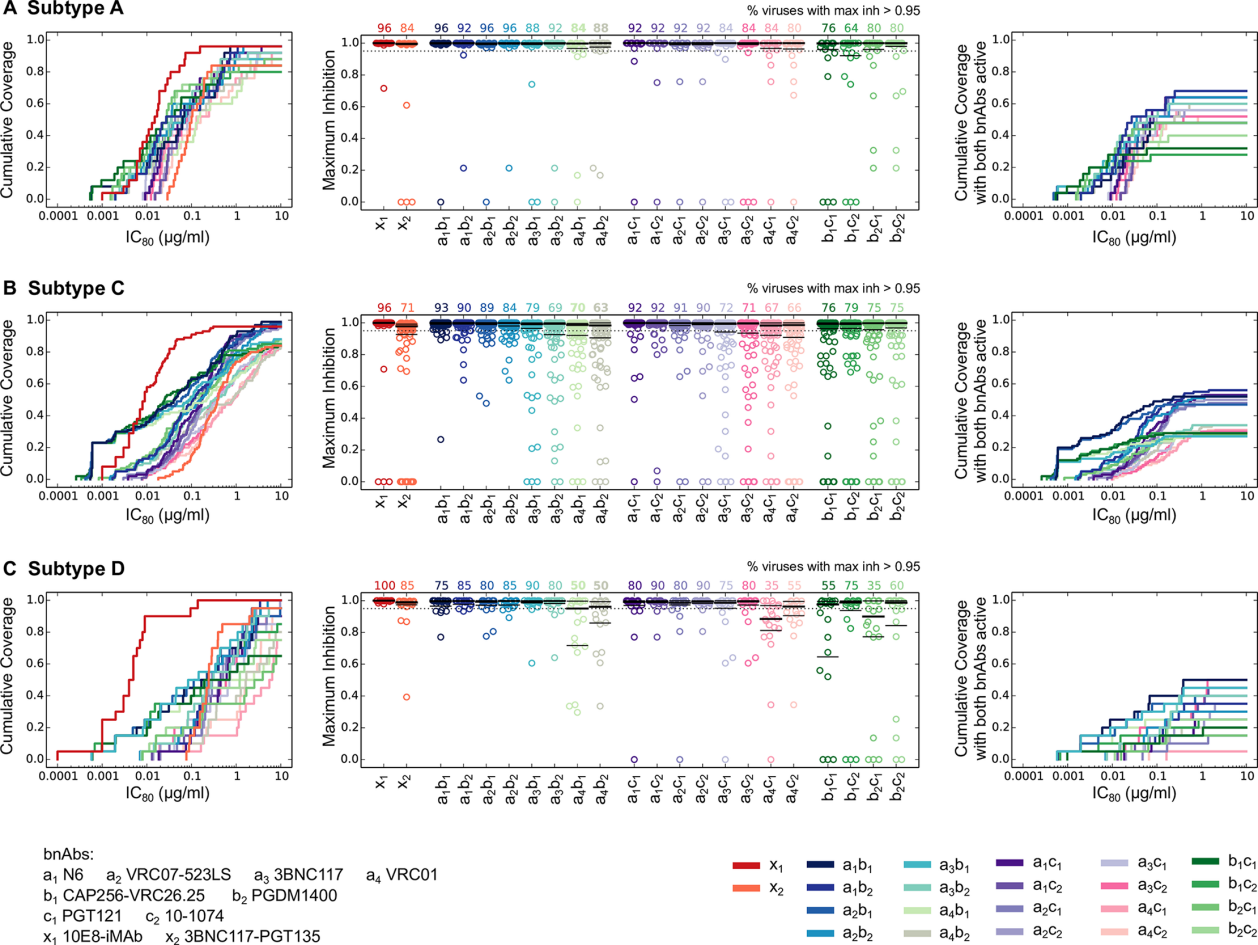
Performance of bispecific Abs and 2 conventional bnAb combinations. (A-C) show the results for subtypes A, C and D, respectively. The left panels show the IC_80_ breadth-potency curves using experimental titers for bispecifics and predicted titers for 2 bnAb combinations. The central panels show fraction maximum inhibition values for bispecific Abs and 2 bnAb combinations against viruses from a given subtype virus panel. Maximum inhibition values are calculated at 5μg/ml for bispecific Abs and at 5μg/ml of each bnAb for 2 bnAb combinations. The right panels show breadth-potency curves for 2 bnAb combination IC_80_ titers by considering only those viruses that were neutralized by both bnAbs with single bnAb IC_80_ < 5μg/ml.

We analyzed three classes of 2 bnAb combinations: CD4bs + V2g, CD4bs +V3g and V2g + V3g. For each subtype the best overall performance across all metrics was observed for CD4bs + V2g combinations, however, the best specific combination was subtype dependent. For subtype A, the best combination was N6 + PGDM1400, with the lowest median IC_80_ titer (0.027 μg/ml, combination titers are reported as total concentration of both bnAbs) (Fig 3A, S2 Table), best IC_80_ coverage (96%), second best coverage of complete neutralization (92%, best was 96%), and best coverage with both bnAbs active (68%). For subtype C, the best combination was N6 + CAP256-VRC26.25, with the second lowest median IC_80_ titer (0.041 μg/ml, best was 0.025 μg/ml), best IC_80_ coverage (99%), best coverage of complete neutralization (93%), and third best coverage with both bnAbs active (52%, best was 56%). For subtype D, the best combination was 3BNC117 + CAP256-VRC26.25, with the most potent median IC_80_ titer (0.114 μg/ml), second best IC_80_ coverage (95%, best was 100%), best coverage of complete neutralization (90%) and second best coverage with both bnAbs active (45%, best was 50%). CD4bs + V3g combinations had somewhat lower performance than CD4bs + V2g combinations. The V2g + V3g combinations had some of the most potent median IC_80_ titers, however, they also had the lowest IC_80_ coverage, completeness of neutralization and especially coverage with both bnAbs active, due to the complementarity between V2g and V3g bnAb neutralization profiles (Fig 1B–1D).

Several of these results were robust to bootstrap variation (S3 Table), however, a few differences were observed. For subtype D, the best combination was predicted to be VRC07-523LS + 10–1074, based on each of its metrics being the best or within 95% bootstrap CI of the best metric. This combination showed the best IC_80_ breadth (100%) and best coverage with complete neutralization (90%) among combinations of two conventional bnAbs for subtype D. In general, we found that several combinations showed metrics that were within 95% bootstrap CI from the best metric for the subtype A and D panels, consistent with their smaller size (n = 25 and 20, respectively). This suggests that larger, representative panels for these subtypes might be needed to accurately inform ranking of 2 bnAb combinations.

As expected, the best 2 bnAb combinations improved performance over individual conventional bnAbs (Fig 2, S2 and S3 Tables) with the same total target concentration (10 μg/ml). Improvements were observed mainly in median IC_80_ titers (2.3–5.2 fold more potent across subtypes, p = 1.5 x 10^−6^–0.035 using Wilcoxon rank sum test on IC_80_ titers) and complete neutralization (0–15% increase, not significant), while IC_80_ coverage was comparable (0–4% increase, not significant). These results reinforce the notion that it is better to combine bnAbs than use the same concentration of a single bnAb. Moreover, given the extent of complete neutralization, passive transfer of the best 2 bnAb combinations has the potential to prevent infection by most diverse strains across all subtypes.

Remarkably, the 10E8-iMAb bispecific performed better than the best 2 bnAb combinations across all subtypes (Fig 3, S2 and S3 Tables), despite a target concentration of 5μg/ml, half of that for the 2 bnAb combinations. 10E8-iMAb was strikingly more potent (1.8–22.8 fold lower median IC_80_ than the most potent 2 bnAb combinations across subtypes; p = 0.0001–0.0256 using Wilcoxon rank sum test; median IC_80_ below the 95% bootstrap CI of the best 2 bnAb combinations for subtypes C and D (S3 Table)), and had higher complete neutralization coverage (3–10%, not significant). For IC_80_ coverage, 10E8-iMAb matched the coverage of the best 2 bnAb combination for subtype A, had 3% lower coverage for subtype C and had 5% higher coverage for subtype D. 10E8-iMAb also has the potential to match 2 bnAb combinations in terms of two independent targets [36], although the strong synergy between the components makes it difficult to measure coverage of 10E8-iMAb with both specificities active.

### Performance of combinations of bispecifics and conventional antibodies

**Fig 4:**
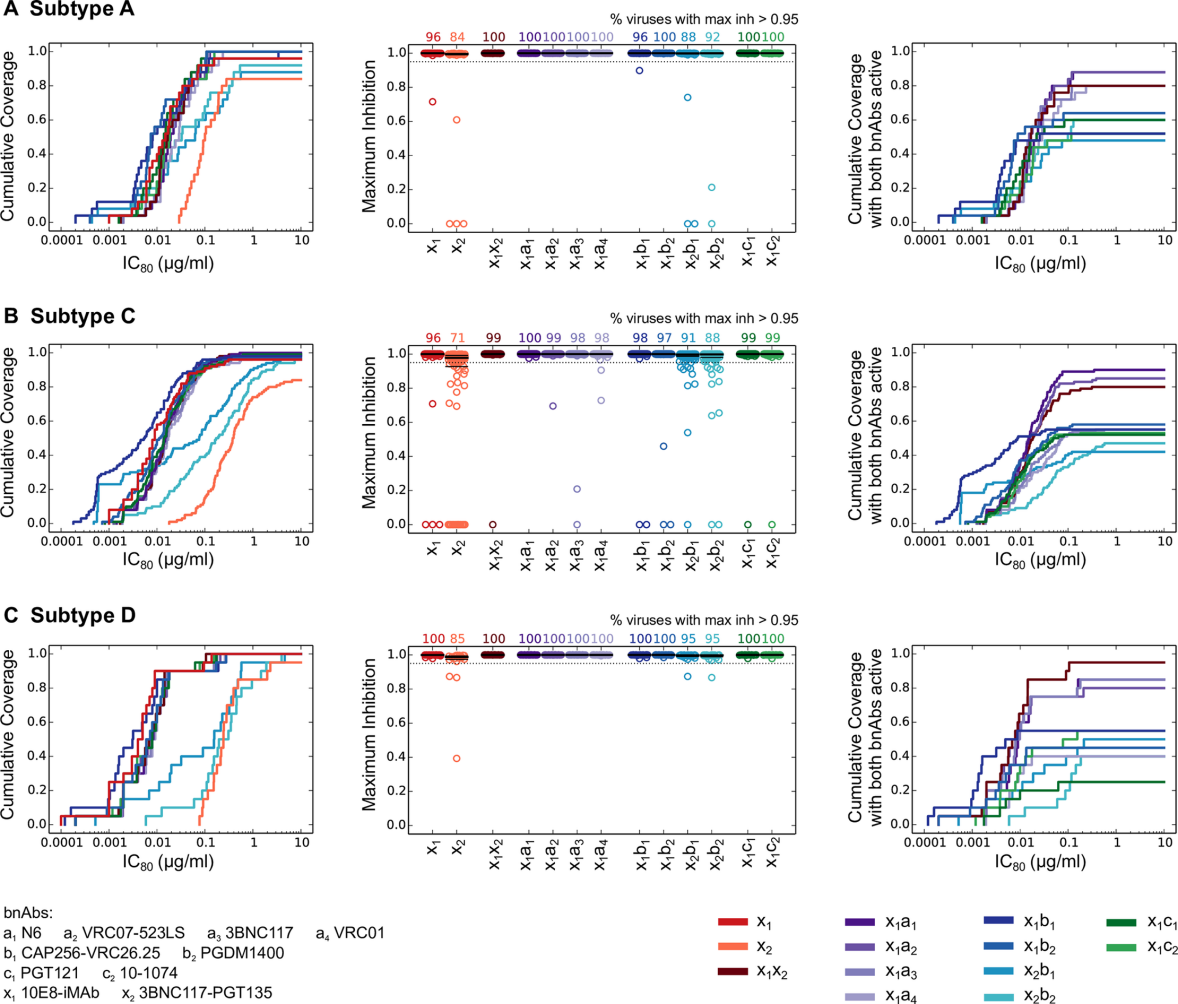
Performance of bispecific Abs and 2 Ab combinations involving bispecifics. (A-C) same as Fig 3A–3C, respectively, except combinations of both bispecific Abs and of the type bispecific + conventional Abs are considered. For combinations of 3BNC117-PGT135 bispecific with bnAbs, only V2 glycan bnAbs are considered to ensure non-overlapping epitope regions with the bispecific.

Building on the impressive performance of 10E8-iMAb, we next investigated the performance of 2 Ab combinations involving bispecifics. We assumed a target concentration of 5μg/ml for each Ab in the combination, and used the Bliss-Hill model to predict the neutralization for the combinations of both bispecifics, and of a bispecific with a conventional bnAb, such that epitope targets are not repeated (combinations of 3BNC117-PGT135 with CD4bs or V3g bnAbs were not considered). As before, we used median IC_80_ titers, IC_80_ coverage, coverage of complete neutralization and coverage with both Abs active as metrics to evaluate performance (Fig 4 and S2 and S3 Tables).

For subtype A, the bispecific combinations with the best overall performance were 10E8-iMAb with VRC07-523LS, N6 or PGDM1400 (S2 Table). The former two combinations showed median IC_80_ titers of 0.016–0.019 μg/ml, completely neutralized all viruses, and neutralized 88% viruses with both Abs active. 10E8-iMAb + PGDM1400 had the lowest median IC_80_ titer (0.007 μg/ml), completely neutralized all viruses, and neutralized 64% viruses with both Abs active. Bootstrap analysis favored 10E8-iMAb + VRC07-523LS as its median IC_80_ fell within the 95% bootstrap CI of 10E8-iMAb + PGDM1400 (S3 Table). For subtype C, the best combination was 10E8-iMAb + N6 with median IC_80_ titer of 0.015μg/ml, complete neutralization of all viruses and neutralization of 90% viruses with both Abs active (S2 and S3 Tables). For subtype D, the best combination was 10E8-iMAb + 3BNC117-PGT135 with the second lowest median IC_80_ titer of 0.007 μg/ml, complete neutralization of all viruses and neutralization of 95% viruses with both Abs active. Across all subtypes, the best combination was 10E8-iMAb + N6, which had relatively less potent median IC_80_ titers than the best, but completely neutralized all viruses and neutralized 85–90% viruses with both Abs active. It was comparable to the combination of both bispecifics for IC_80_ potency and coverage of complete neutralization, however, it showed higher coverage with both Abs active for subtypes A and C (8 and 10%, respectively) (for subtype D it showed 10% lower coverage with both Abs active) (S2 Table). The differences in coverage with both bnAbs active for subtypes A and D were not robust to bootstrap (S3 Table).

The best combinations involving bispecifics performed better than either 10E8-iMAb alone or the best 2 conventional bnAb combinations. The improvements were complete neutralization of all viruses (10E8-iMAb and best 2 bnAb combinations incompletely neutralized 0–10% viruses across subtypes) and substantial increase in coverage with both Abs active (20–50% increase over best 2 bnAb combinations; not significant for subtype A and p = 2.9 x 10^−9^ and 0.001 for subtypes C and D, respectively, using Fisher’s exact test; higher than 95% bootstrap CI for best 2 bnAb combinations for subtypes C and D (S3 Table)). The latter could be important as viral resistance can emerge in chronically infected mice treated with 10E8-iMAb [36]. In such cases, combinations involving bispecifics may be advantageous as bispecific + conventional Ab combinations effectively have three independent targets and the combination of bispecifics have four independent targets. In terms of potency, the overall best bispecific combinations sometimes had less potent median IC_80_ titers than 10E8-iMAb alone because of the conventions of equal concentration of Abs in the combination and combination IC_80_ titers reported as the total Ab concentrations. However, V2g bnAbs combined with 10E8-iMAb showed more potent IC_80_ titers than for 10E8-iMAb alone (S2 Table).

### Modeling antibody mediated protection *in vivo*

The protective effect of passively transferred Abs in preventing SHIV infections has been shown in macaques [13, 15–18, 21, 22]. While these studies highlight the potential of bnAbs for protection, no strategy exists to predict *in vivo* protection using the *in vitro* neutralization profile of a given Ab against a given challenge virus. Here we begin to address this question by modeling Ab mediated protection using data from a repeated, low-dose SHIV challenge macaque study by Gautam et al. [21]. In this study, a single injection of 20 mg/kg of one monoclonal antibody, 10–1074, 3BNC117, VRC01 or the longer half-life variant of VRC01 (VRC01-LS), was given to six macaques per Ab group, and nine macaques were used as controls. Each animal was challenged weekly with a low-dose SHIV_AD8-EO_ inoculum by the intrarectal route until they got infected. The *in vivo* protective effect of each Ab was significantly higher than control, and modeling of protection as a function of Ab concentration showed that the more potent the Ab against SHIV_AD8-EO_, the higher the protective effect.

To explore whether differences in the *in vivo* protective effect between Ab groups could be predicted using *in vitro* potency of Abs, we modeled *in vivo* protection as a function of *in vitro* neutralization corresponding to the Ab titers at the time of each challenge. We predicted the fraction neutralization of the SHIV_AD8-EO_ pseudovirus using measured or interpolated *in vivo* concentrations of Abs, and the *in vitro* pseudovirus IC_50_ and IC_80_ Ab titers reported in Gautam et al (Methods). We transformed the fraction neutralization to instantaneous inhibitory potential (IIP) [53] as our dependent variable, since it provided better fits. IIP is defined as -Log_10_(1.0 –fraction neutralization), and measures the Log_10_ reduction in a single round of infection. We modeled the binary variable “protected” or “not protected” for each challenge as a function of IIP using modified logistic regression models, with model parameters determined using likelihood maximization (Methods, S1 Text). These modified models account for the baseline probability of infection for the low-dose challenge by having a scale parameter for the maximum probability of infection (p_0_) that is fit using experimental data. We compared the fits of the experimental data using two models: a) with the same parameters across all Abs (3 parameters in total), and b) with different parameters for all Abs (9 parameters in total) (S1 Text). Using model selection criteria the maximum-likelihood model with same parameters across Abs was better (difference in AIC = 1.98, and in BIC = 24.90), and both models provided similar likelihoods (p = 0.12 using likelihood ratio test). We tested the goodness of fit of the model with same parameters by using the Hosmer-Lemeshow test (Methods), which estimates the statistical significance for rejecting the hypothesis that the fitted model is the true model [54]. For the above model, we obtained p = 0.9871, which indicates a good fit of our model to the experimental data. Thus, these results together suggest that the 4 Abs from Gautam et al. provided similar *in vivo* protection as a function of IIP, and that the differences in protection in this study may be explained by differences in potency and pharmacokinetics of the Abs.

**Fig 5:**
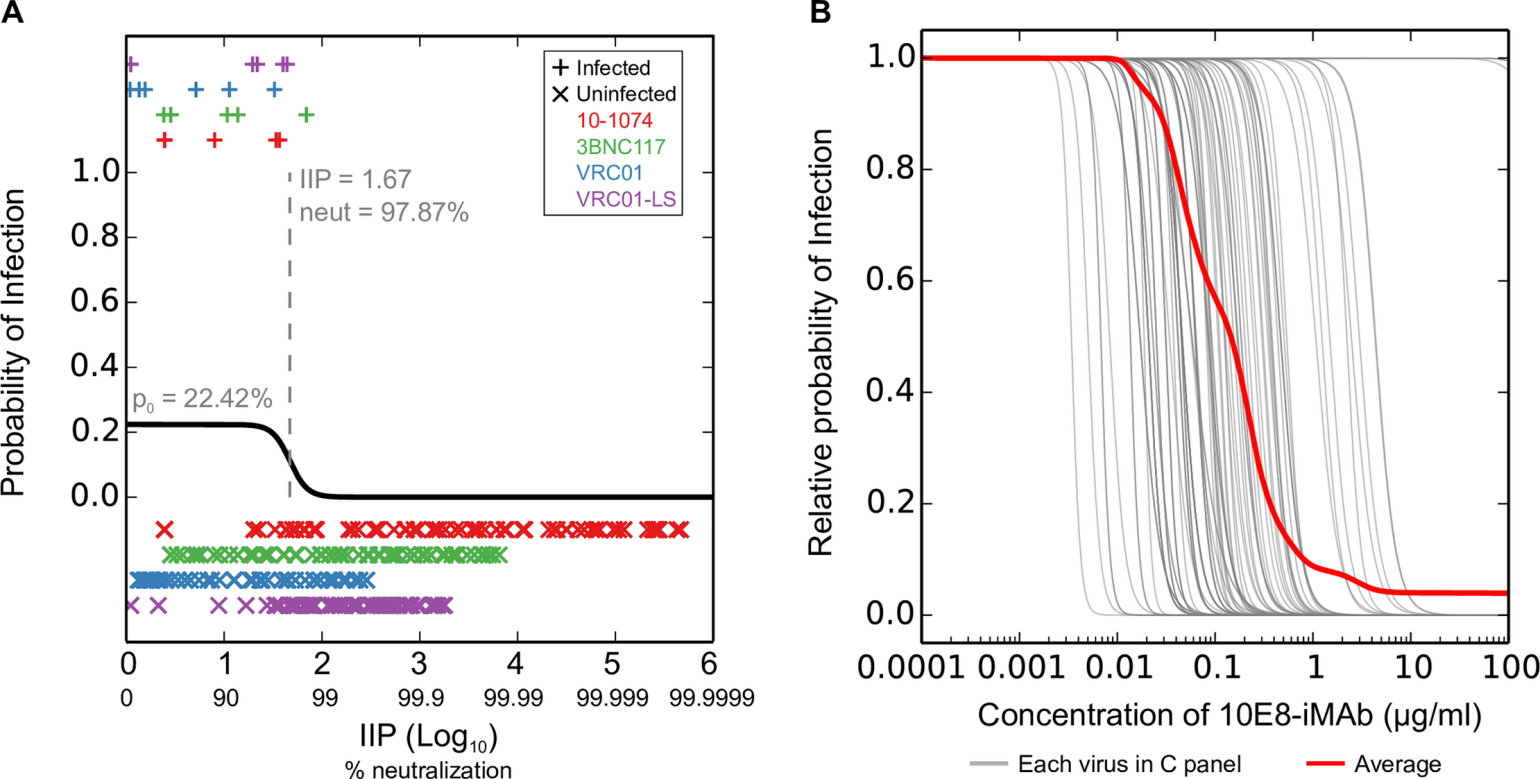
Modeling *in vivo* protection as a function of *in vitro* neutralization. (A) The maximum likelihood model for the *in vivo* probability of infection as a function of IIP using data from the low-dose, repeated SHIV challenge macaque study [21] is shown in black line. This model had the same parameters across all Abs. The IIP values at the time of challenge when animals were infected are shown on top with ‘+’ and when animals were protected are shown below with ‘x’. Data for animals from different Ab groups are shown separately with different colors, and data for control animals (9 out of 33 challenges resulting in infection) are not shown. (B) The relative probability of infection as a function of concentration of the bispecific 10E8-iMAb against each clade C panel pseudovirus (grey lines) and the average across the panel viruses (red line).

The maximum likelihood model with same parameters for Abs is shown in Fig 5A. This model has a baseline probability of infection for the low-dose SHIV challenge of 22.42%, consistent with the 9 out of 33 challenges resulting in infection of control animals (p = 0.53 using binomial test). The probability of infection was significantly negatively correlated with IIP (p = 6.35x10^-12^, using likelihood ratio test, S1 Text). However, the protective effect of Abs was seen at high fraction neutralization, with < 5% relative protection (defined as 100 - % relative probability of infection) for < 96.1% neutralization. Above this, the protection probability was predicted to have a sharp transition, with 50% relative protection for 97.9% neutralization and > 95% relative protection for > 98.8% neutralization. To estimate the robustness of the model parameters, we simulated 1,000 bootstrap randomizations (Methods, S1 Fig); the estimated parameters for baseline probability of infection (median = 23.68%, interquartile range = 22.37–25.29%) and neutralization for 50% relative protection (median = 97.77%, interquartile range = 97.18–98.05%) were robust and close to the best-fit model. However, the slope of the scaled logistic curve (median = 11.97, interquartile range = 7.64–37.93) showed relatively higher variation, with ~22% realizations showing slopes >100, suggesting that the slope of the best-fit model might show some dependence on the exact data used.

**Fig 6:**
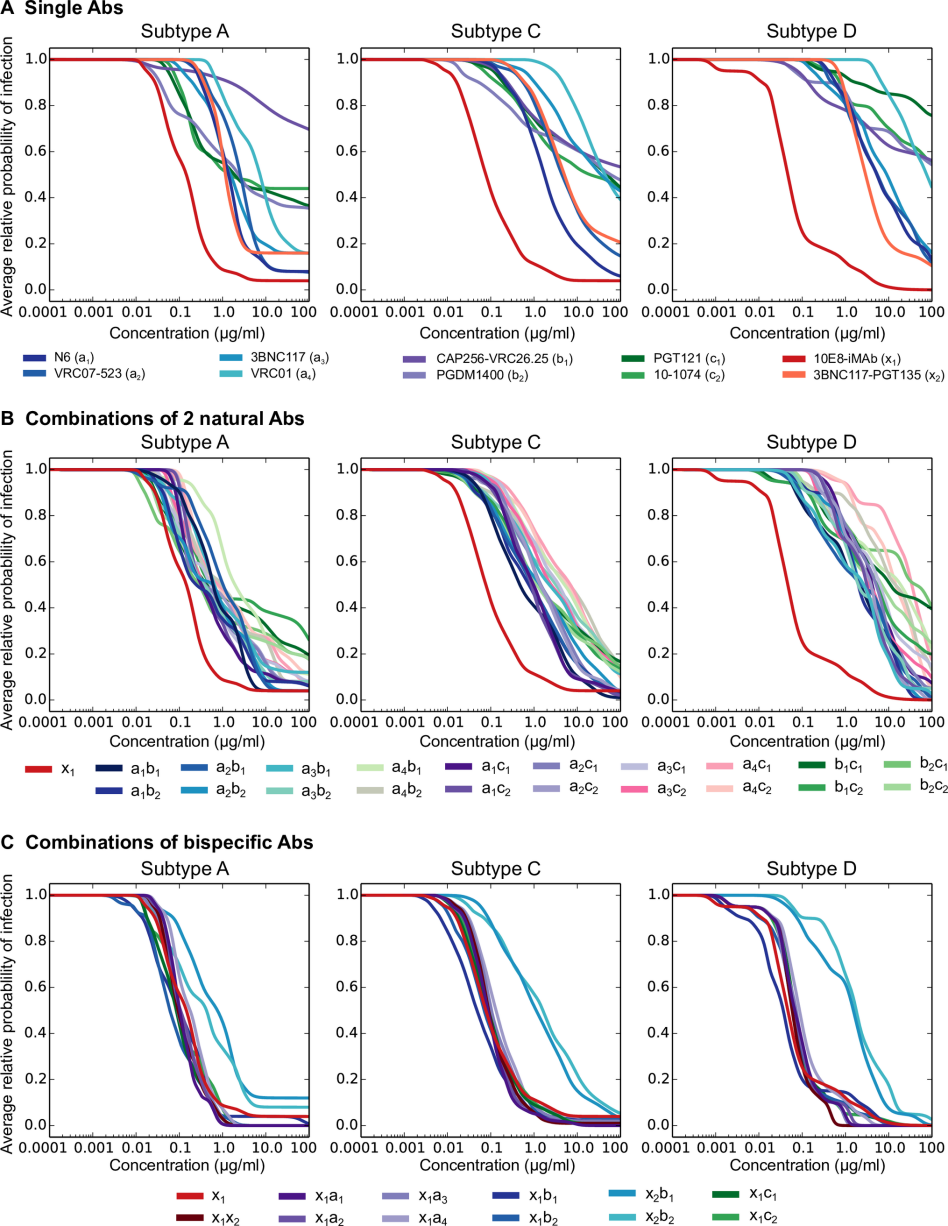
Predicted *in vivo* protection for individual Abs and combinations. The average relative *in vivo* probability of infection predicted using above modeling is shown for single Abs (A), combinations of two conventional Abs (B) and combinations with one or two bispecific Abs (C) for the subtype A (left), C (middle) and D (right) panels. The curves show the relative probability of infection at a given concentration averaged over pseudoviruses from each panel. For Ab combinations, the total concentration of both Abs is shown.

We used the above model to predict the *in vivo* protection offered by Abs and Ab combinations in this study. Since the baseline rates of human HIV-1 infections are much lower than those estimated for the low-dose SHIV challenge above, we used the relative probability of infection to model the performance of Abs and Ab combinations. We calculated IIP for Abs and combinations using the Bliss-Hill model (Methods) for a range of concentrations and predicted the relative probability of infection for each virus and the average relative probability of infection for a panel by averaging over all viruses in the panel (Fig 5B). The average relative probability of infection as a function of concentration for Abs and Ab combinations are shown in Fig 6 and numerical values at the target concentrations of 5 or 10μg/ml are reported in S2 Table. The results from bootstrap simulations of each pseudovirus panel are shown in S2–S4 Figs and bootstrap medians and 95% CI are reported in S3 Table.

Among single Abs, 10E8-iMAb had the lowest average relative probability of infection of 3–5% across subtypes at 5μg/ml. It was significantly better than the next best, N6, with 11–39% across subtypes at 10μg/ml (p = 7.2x10-12–0.0006 using Wilcoxon rank sum test on relative probability of infection values for each virus in a panel) (Fig 6A, S2 Table). The differences between 10E8-iMAb and other bnAbs were significant for subtypes C and D using 95% bootstrap CI (S3 Table). VRC07-523LS was comparable to N6, but other single Abs were predicted to have limited protection across subtypes. VRC01 showed 40% average relative probability of infection for subtype A, but 78% for subtype C and 85% for subtype D. Similarly, V2g and V3g bnAbs had minimum average relative probability of infection of 40–63% across subtypes. 3BNC117-PGT135 showed intermediate performance with 16–50% average relative probability of infection across subtypes.

The best 2 conventional bnAb combinations showed 4–18% average relative probability of infection across subtypes and were different for each subtype: N6 + CAP256-VRC26.25 for subtype A, N6 + PGT121 for subtype C and 3BNC117 + CAP256-VRC26.25 for subtype D (Fig 6B, S2 and S3 Tables). While N6 + CAP256-VRC26.25 showed slightly better protection than 10E8-iMAb for subtype A (0.27% lower average relative probability of infection, not significantly different using bootstrap analysis (S3 Table)), 10E8-iMAb was better than the best 2 bnAb combinations for subtypes C (4.3% lower average probability of infection, p = 0.06, Wilcoxon rank sum test) and D (14.7% lower average probability of infection, p = 0.005 Wilcoxon rank sum test and significantly different using 95% bootstrap CI (S3 Table)), in spite of having half the total target concentration.

Combinations of 10E8-iMAb with other Abs reduced the average probability of infection across all subtypes (Fig 6C, S2 and S3 Tables). 10E8-iMAb + N6 showed the best protection with average relative probability of infection of < 1.3 x 10^−7^% for subtypes A and D and of 0.54% for subtype C, a significant improvement over 10E8-iMAb (p = 3.6 x 10^−18^–0.002 across subtypes using Wilcoxon rank sum test, and significantly different using 95% bootstrap CI (S3 Table)). 10E8-iMAb + 3BNC117-PGT135 was next with an average relative probability of infection of 7.2 x 10^−8^–1% across subtypes. These results raise the possibility that 10E8-iMAb combinations with N6 or 3BNC117-PGT135, may be very effective at preventing almost all subtype A, C and D infections.

## Discussion

Given the absence of effective Ab-based vaccines against HIV-1, and the difficulties in adherence to antiretroviral drug PrEP, passive transfer of Abs is a promising alternative prophylactic modality [4, 12]. Here, we characterized the potential of the most clinically advanced conventional and engineered Abs, and their two antibody combinations, to prevent infections in sub-Saharan Africa, by analyzing *in vitro* neutralization metrics and modeling of *in vivo* protection.

Modeling of data from a macaque challenge study highlighted the potential challenges for Ab-mediated *in vivo* protection. In particular, the protective effect of Abs was observed only beyond 96% neutralization, suggesting that near-complete neutralization, even beyond our assumed cutoff of 95% neutralization, might be important for consistent *in vivo* protection. Since the number of infectious challenges was relatively small in this dataset (n = 33 out of 337 total), it is possible that our modeling would miss low levels of protection at low neutralization, rendering our estimates to be conservative. However, our result is consistent with previous findings that plasma ID_50_ titers of ~40–200, using different bnAbs, can protect against SHIV challenges with different viruses, doses and routes of challenge [15, 17, 18, 23, 55]. These serum titers correspond to Ab concentrations of 40–200 times IC_50_ titers, which assuming an average neutralization curve with slope = 1 [38, 56], yield ~97.5–99% *in vitro* neutralization [38, 56]. Nonetheless, we found that 10E8-iMAb, which targets MPER and host-cell CD4, alone and in combination with other Abs, can still meet these stringent requirements of near complete neutralization.

It is not clear how applicable for human infections is the above modeling of *in vivo* protection using macaque data due to several potential differences. First, even the low-dose SHIV challenge is much more infectious than typical human sexual transmissions (~30% baseline infection rate versus ~0.1–1% [57, 58]). Second, our model was derived using data on the single subtype B SHIV_AD8-EO_ challenge, and whether it will hold true for different viruses with different baseline infection rates is not clear. Third, while we found that the four bnAbs studied in Gautam et al. could be modeled using the same parameters, it is not clear whether this will apply for all the Abs/combinations in this study. Fourth, our model does not account for any potential contribution of effector functions or other non-neutralizing antibody functions to *in vivo* antibody mediated protection; although, the good fits of experimental data suggest that these effects could be minor in comparison to neutralization. Nonetheless, our modeling clarifies the relationship between *in vitro* neutralization and *in vivo* protection in macaques, and introduces a novel statistical framework to explore the question whether or not there are universal features of Ab protection, as future human and animal studies are undertaken.

The use of *in vitro* neutralization metrics to inform the *in vivo* performance of Abs intrinsically has limitations. Two issues that may impact this study are that the *in vitro* assays used here were based on pseudoviruses grown in 293T cells, which can result in bnAb-specific neutralization differences relative to molecular clones grown in PBMCs [38, 59]. As the latter are more relevant *in vivo*, such differences could impact the relative ranking of Abs and Ab combinations obtained using pseudovirus-based *in vitro* metrics. Another important factor missing from our analysis is *in vivo* Ab stability, which can vary between Abs and can impact the choice of optimal Abs and Ab combinations for clinical efficacy.

The variable performance of single bnAbs highlights the difficulty of meeting the challenges in the prevention setting. While V2 and V3 glycan bnAbs were some of the most potent, they potently neutralized less than 50% of the pseudoviruses tested (IC_80_ < 0.1 μg/ml) (Fig 2), and had low coverage of complete neutralization and predicted relative protection *in vivo* across subtypes. The CD4bs bnAbs improved coverage of complete neutralization and relative protection; however, this performance was observed for some but not all subtypes. For example, VRC01 efficacy was predicted to be lower for subtypes C and D, and higher for subtype A, highlighting the importance of considering viral subtypes among breakthrough cases in the phase 2b VRC01 Antibody Mediated Prevention (AMP) clinical trials [60]. Given the limitations discussed above, our models may not be predictive of outcomes in a clinical setting, however, data from clinical trials will be invaluable in understanding how predictive *in vitro* neutralization can be of *in vivo* protection in human infections, and will help refine the models developed here.

Our results indicated that combinations of 2 conventional bnAbs would substantially improve the performance over the single bnAbs, even with the same total concentration. This improvement was most notable for complete neutralization and relative probability of infection. The best combinations consisted of one CD4bs and one V2 glycan bnAb, however, the optimal combination differed for each subtype. Overall, N6 with CAP256-VRC26.25 or PGDM1400 showed best performance across subtypes, although their performance was limited for subtype D. Thus, at the total target concentration of 10 μg/ml, even combinations of 2 bnAbs might be insufficient for prevention of some infections, across subtypes prevalent in sub-Saharan Africa. Nonetheless, 2 bnAb combinations are predicted to be highly preferable over single bnAbs. Since combinations target two independent targets, they will also increase the coverage of the donor quasispecies diversity, as chronically infected donors can have viruses resistant to single bnAbs.

Bispecific Abs offer a way to increase breadth and potency by combining different paratopes in a single molecule, thus overcoming some of the above challenges. The bispecific 3BNC117-PGT135 was comparable to the best conventional bnAbs, but was outperformed by some 2 bnAb combinations. However, we found that 10E8-iMAb showed superior performance over all single Abs and, remarkably, even over all combinations of two conventional bnAbs. This performance was found across subtypes at the lower assumed concentration of 5μg/ml, half that of bnAbs/bnAb combinations. 10E8-iMAb also has two independent components, both of which are individually quite broad, with significant synergy between them in the context of the bispecifics [36]. Still, 10E8 resistance mutations in Env allowed escape in most chronically infected mice treated with 10E8-iMAb [36], suggesting that combining 10E8-iMAb with other Abs can improve coverage of within-host quasispecies and reduce the opportunity for emergence of resistance. Indeed, our modeling indicated that 10E8-iMAb when combined with N6 or 3BNC117-PGT135 showed 80–95% coverage with both Abs active and very low average relative probability of infection (<0.6%) across subtypes. Since 10E8-iMAb does not retain Fc effector functions such as antibody dependent cellular cytotoxicity (ADCC), combining 10E8-iMAb with Abs with ADCC activity might provide an additional advantage. It is not clear how important Fc effector functions might be for sterilizing protection relative to neutralization, however previous studies suggest a beneficial role, as such functionalities may help clear infections as they are beginning to disseminate [15, 20, 61]. Thus, combining 10E8-iMAb with potent Abs like N6 or 3BNC117-PGT135 substantially improves the already impressive predicted potential of 10E8-iMAb to prevent HIV-1 infections in sub-Saharan Africa.

## Materials and methods

### Study design

This study was designed to analyze the potential of passively transferred Abs and Ab combinations to prevent HIV-1 subtype A, C and D infections. We collected *in vitro* neutralization data for 10 conventional and bispecific Abs against a total of 145 pseudoviruses and used computational modeling on these data to predict neutralization data for Ab combinations. We analyzed these data to compare the performance of Abs and Ab combinations. We also modeled Ab-mediated *in vivo* protection using data from a published macaque challenge study and used this to predict the relative *in vivo* protection offered by Abs and Ab combinations in this study.

### Viruses and antibodies

Panels of HIV-1 Env pseudoviruses representative of clades A (n = 25), C (n = 100), and D (n = 20) were utilized to assess the breadth and potency of bnAb neutralizing activity. The clade C virus panel is a subset of the larger 200 virus panel of early/acute isolates previously described [49, 50]. The clade A and D pseudoviruses are derived from isolates from HIV-infected patients from sub-Saharan Africa obtained as part of the CAVD Comprehensive Antibody Vaccine Immune Monitoring Consortium’s (CAVIMC) Standard Virus Panel Project and exhibit a Tier 2 neutralization phenotype; information about these viruses is presented in S1 Table. Env pseudovirus stocks were generated by transfection of 293T/17 cells (American Type Culture Collection (ATCC), Manassas, VA) as previously described [62]. While the pseudoviruses panels used here are a resource shared throughout the field, developed with the intention of being representative of circulating viruses, still they are subject to bias. Two documented issues suggest they may not be fully representative of the levels of resistance that would be encountered in a prevention trial. In particular, HIV-1 is diversifying over time, and becoming increasingly resistant at the population level, measurable on the time scale of decades [49, 63]. As neutralization panels take years to develop, the original samples from which the pseudoviruses were derived were often sampled 10–20 years ago. In addition, transmitted-founder viruses tend to be more resistant to antibodies, and many of the viruses in our panels were sampled during chronic infection [49, 64].

The panel of 10 broad and potent monoclonal antibodies tested here was selected based on their current use in passive infusion clinical trials, or considered advanced candidates for clinical development. Cloned human antibodies were generated in the laboratories of M. Nussenzweig (3BNC117, 10–1074), D. Burton (PGT121, PGDM1400), M. Connors (N6), or at the NIH Vaccine Research Center (VRC01, VRC07-523, CAP256-VRC26.25). Engineered bispecific antibodies were generated in the laboratories of D. Ho (10E8-iMAb) and J. Ravetch (3BNC117-PGT135).

### Neutralization assays

Neutralizing antibody titers were determined using a luciferase-based reporter assay in TZM.bl cells as previously described [65, 66]. Starting concentrations of individual Abs ranged from 10–50 ug/ml depending on available supply at the time of testing. All Abs were serially diluted seven times using a 5-fold titration series. All assays were performed in a laboratory meeting GCLP standards. Data for 3BNC117, 10–1074, VRC01, VRC07-523LS and CAP256-VRC26.25 for some A and D pseudoviruses were used from previous studies [23, 29, 36, 43, 45, 67].

### Modeling of neutralization by Abs and Ab combinations

We used the previously developed Bliss-Hill model [30] to predict the IC_80_ and fraction maximum inhibition values for 2 Ab combinations, using the web tool, CombiNAber (https://www.hiv.lanl.gov/content/sequence/COMBINABER/combinaber.html). The individual Ab IC_50_ and IC_80_ experimental data was the input, and a target concentration of 5μg/ml of each Ab in the combination was used. We predicted incomplete neutralization values for single Abs against panel viruses by assuming a Hill curve (f(c) = c^m^ / (IC_50_^m^ + c^m^), where f is fraction neutralized, c is concentration of Ab and m = log(4) / [log(IC_80_)–log(IC_50_)]), as implemented in CombiNAber.

### Bootstrap analyses

To understand variation of the metrics studied here with respect to finite sampling, we used a bootstrapping approach. 1,000 bootstrap replicates for each pseudovirus panel were generated by randomly sampling viruses with replacement, with each replicate matching the size of the pseudovirus panel. For each bnAb/combination and for each subtype, each metric was evaluated for these bootstrap replicates and the medians and 95% CI were calculated (S3 Table).

### Statistical analyses

We performed statistical comparisons using packages implemented in the Stats module from SciPy [68]. Non-parametric tests were preferred and two-sided p-values are reported.

### Modeling of *in vivo* protection as a function of in vitro IIP

We used the *in vivo* Ab concentrations reported in Gautam et al. [21] to obtain concentration at the time of each challenge for each macaque. If the concentration at the time of challenge was not reported, we interpolated the concentration assuming a log-linear decay of Ab concentration between the timepoints immediately before and after the challenge with reported concentrations; or extrapolated the concentration by using the previous two time points with reported concentrations. To match the experimental data, a minimum threshold of 0.1 μg/ml was used for predicted concentrations. We used Hill curves to predict the fraction neutralization at a given concentration of an Ab using the IC_50_ and IC_80_ titers against SHIV_AD8-EO_ pseudovirus from Gautam et al., and transformed this to IIP using IIP = -Log_10_(1.0 –fraction neutralization).

We used modified versions of logistic regression models to model the probability of infection *in vivo* as a function of IIP (S1 Text). The best model with same parameters across Ab groups was: p(*x*) = p_0_ / [1 + exp(*a x* + *b*)], where *x* is IIP and parameters *p*_*0*_, *a* and *b* were fixed using maximum likelihood on data from all challenges in all animals across control and Ab groups. We used the SciPy package for constrained optimization algorithm ‘L-BFGS-B’ [68, 69] to estimate maximum likelihood parameters, which were *p*_*0*_ = 0.2242, *a* = 11.2994 and *b* = -18.8970.

The goodness-of-fit for the above model was tested using the Hosmer-Lemeshow test [54], as implemented in R. Given the size of our dataset (337 data points), we used 10 groups for the Hosmer-Lemeshow test as recommended by Paul et al. [70]; however, our result was robust when we used 5–15 groups. Likelihood ratio tests were performed to compare models and estimate significance of parameters as reported in S1 Text. For bootstrap simulations, we generated 1,000 realizations using random sampling of observed data with replacement to obtain the same number of infected and uninfected data points as in the observed data, and fit the above scaled logistic model to each bootstrap realization.

For prediction of *in vivo* relative protection of Abs and Ab combinations from this study, the above model was used with IIP values at a given concentration predicted either using Hill curves for single Abs or using Bliss-Hill model for Ab combinations as implemented in CombiNAber [30]. Average relative protection for a virus panel was obtained by averaging over relative protection for all viruses in the panel at a given concentration of Ab or Ab combination.

## Supporting information

S1 Text Details for modeling of *in vivo* protection as a function of *in vitro* IIP. (DOCX)

S1 Fig Bootstrap robustness of models of Ab-based *in vivo* protection. We simulated 1,000 bootstrap realizations and obtained best-fit scaled logistic models for probability of infection as a function of IIP for each realization (Methods). (A) The scaled logistic curves for bootstrap realizations are shown in translucent black and that using the observed data is shown in solid red. (B-D) Histograms of best-fit model parameters for each bootstrap realization are shown using grey bars, and those for the observed data are shown using red vertical lines.
(TIF)

S2 Fig Bootstrap variation of predicted *in vivo* protection of individual Abs and combinations against subtype A pseudovirus panel. Each panel shows the average relative *in vivo* probability of infection as a function of concentration for individual Abs and combinations using the full subtype A pseudovirus panel data (red dashed curves) and using 1,000 bootstrap replicates (Methods). The bootstrap median curves are shown with black lines, the interquartile range (25–75 percentiles) at each concentration shown using dark grey shaded regions and the 95% confidence intervals shown using light grey shaded regions.
(TIF)

S3 Fig Bootstrap variation of predicted *in vivo* protection of individual Abs and combinations against subtype C pseudovirus panel. Same as S2 Fig, except using subtype C pseudovirus panel.
(TIF)

S4 Fig Bootstrap variation of predicted *in vivo* protection of individual Abs and combinations against subtype D pseudovirus panel. Same as S2 Fig, except using subtype D pseudovirus panel.
(TIF)

S1 Table Virus information for subtype A and D pseudovirus panels.
(XLSX)

S2 Table Summary of metrics used to evaluate performance for all individual Abs and Ab combinations against all subtypes. (XLSX)

S3 Table Bootstrap median and 95% confidence intervals for metrics used to evaluate performance for all individual Abs and Ab combinations against all subtypes. (XLSX)

S1 Data **IC_50_ and IC_80_ titers for individual Abs against subtype A and D panels**.
(XLS)

S2 Data IC_50_ and IC_80_ titers for individual Abs against subtype C panel. (XLSX)
