# Supplementary material for: Potential of conventional & bispecific broadly neutralizing antibodies for prevention of HIV-1 subtype A, C & D infections
Source: PLoS Pathog. 2018 Mar 5;14(3):e1006860. doi: 10.1371/journal.ppat.1006860 (PMC5854441; doi:10.1371/journal.ppat.1006860)
Supplement: S1 Text — (DOCX) [file ppat.1006860.s001.docx]

**Text S1: Details for modeling of *in vivo* as a function *in vitro* IIP**

The conventional logistic regression model is p(*x*) = 1 / [1 + exp(*a x + b*)], where p is the probability of an event (in our case infection from a challenge), *x* is the dependent variable and *a*, *b* are parameters to be determined using maximum likelihood. To account for the fact that the low-dose SHIV challenge used in Gautam et al. *(21)* had a baseline probability of infection of < 100% (estimated to be ~ 27% using 9 out 33 infected challenges for the control group), we decided to use the following modified logistic regression model: p(*x*) = *p_0_* / [1 + exp(*a x + b*)], where the parameter *p_0_* governs the maximum value of probability and is fit using data. For our modeling, the dependent variable *x* is IIP and best-fit parameters were found using maximum likelihood on data from all animals from all antibody (Ab) and control groups. This baseline model assumes that each antibody has the same parameters. We further tested variations of this model shown below to understand: a) whether there is statistical support to claim that each Ab has the same parameters, and b) the significance of parameters found in the above baseline model using likelihood ratio tests.

| **Mo-del #** | **Equation** | **# of param-eters** | **Maximum likelihood parameters** | **Log Likelihood** | **AIC (BIC*)** |
| --- | --- | --- | --- | --- | --- |
| M0 | p(*x*) = *p_0_* / [1 + exp(*a x + b*)] | 3 | *p_0_*=0.2242, *a*=11.2994, *b*=-18.8970 | -52.4129 | 110.83 (122.29) |
| M1 | For Ab group *i*:  p(*x*) = *p_0_* / [1 + exp(*a_i_ x+b_i_*)]  For control group:  p(*x*) = *p_0_* | 9 | *p_0_* = 0.2313  For 10-1074:  *a* = 161.7515, *b* = -259.2022  For 3BNC117:  *a* = 161.8905, *b* = -303.6958  For VRC01:  *a* = 426.5588, *b* = -668.4829  For VRC01-LS:  *a* = 257.4272, *b* = -425.8223 | -47.4036 | 112.81 (147.19) |
| M2 | p(*x*) = 1 / [1 + exp(*a x + b*)] | 2 | *a* = 1.2349, *b* = 0.7328 | -56.6613 | 117.32 (124.96) |
| M3 | p(x) = *p_0_* | 1 | *p_0_* = 0.0979 | -78.1960 | 158.39 (162.21) |

* Number of total data points (challenges) = 337

Comparison of models M0 and M1 using likelihood ratio test suggested that M0 has similar likelihood as M1 (p = 0.1239 using difference of 6 parameters). Furthermore, M0 had lower AIC and BIC than M1. These results suggested that there is no statistically significant support for differences between Abs and all Abs can be assumed to provide similar protection as a function of IIP. Similarly, comparison of models M0 and M2 indicated that *p_0_* is significantly less than 1.0 (p = 0.0036, likelihood ratio test), thus suggesting that our modified baseline model (M0) is a significantly more appropriate model than the conventional logistic regression model for *in vivo* protection as a function of IIP. Comparison of models M0 and M3 indicated that contribution of IIP to probability of infection is significant (p = 6.4 x 10^-12^, likelihood ratio test). This result suggests that probability of infection is significantly negatively dependent on the *in vitro* fraction neutralization afforded by the Abs at *in vivo* concentrations at the time of challenge.
