# Supplementary figures and images for: Potential of conventional & bispecific broadly neutralizing antibodies for prevention of HIV-1 subtype A, C & D infections
Source: PLoS Pathog. 2018 Mar 5;14(3):e1006860. doi: 10.1371/journal.ppat.1006860 (PMC5854441; doi:10.1371/journal.ppat.1006860)

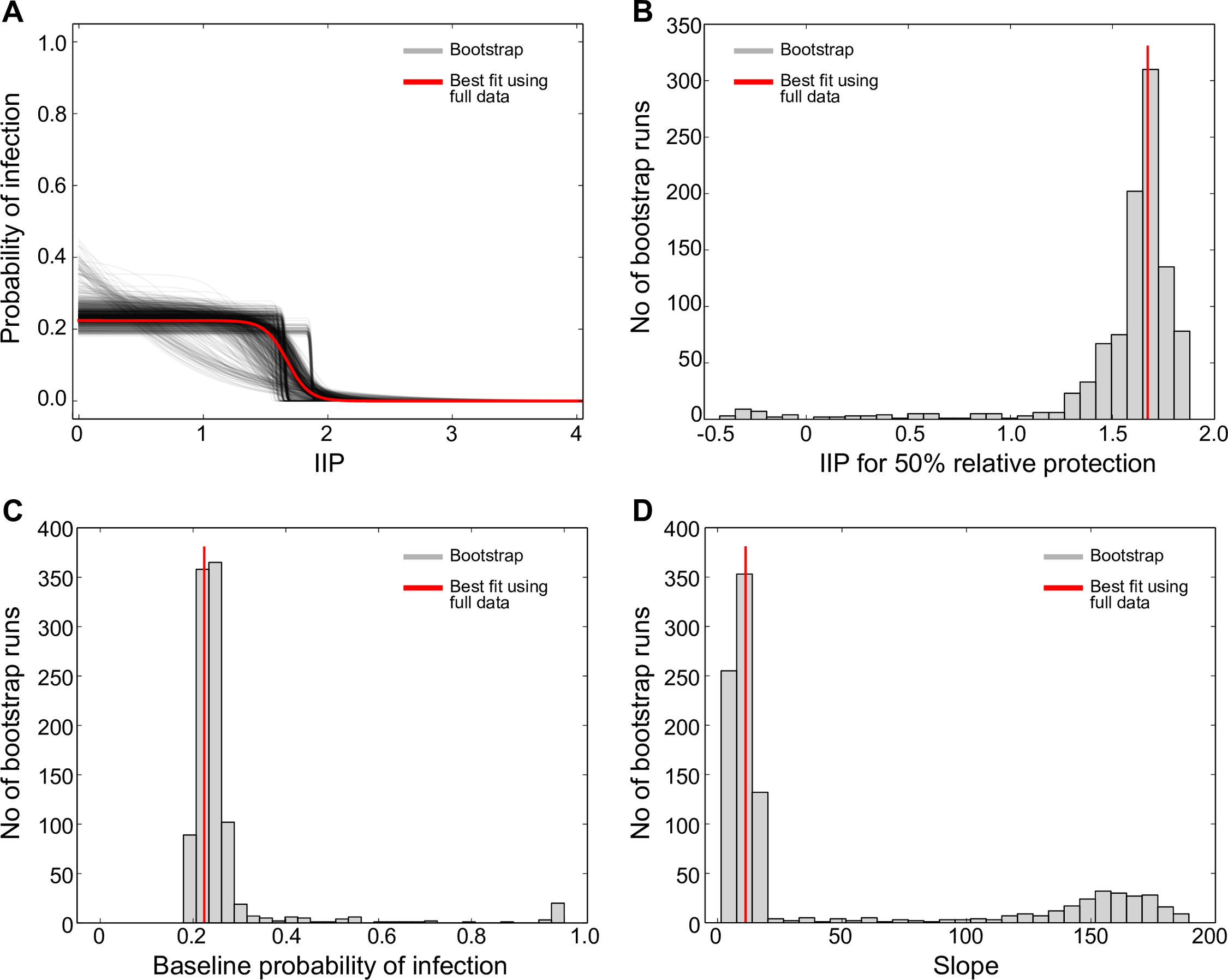

Supplement: S1 Fig — We simulated 1,000 bootstrap realizations and obtained best-fit scaled logistic models for probability of infection as a function of IIP for each realization (Methods). (A) The scaled logistic curves for bootstrap realizations are shown in translucent black and that using the observed data is shown in solid red. (B-D) Histograms of best-fit model parameters for each bootstrap realization are shown using grey bars, and those for the observed data are shown using red vertical lines. (TIF) [file ppat.1006860.s002.tif]

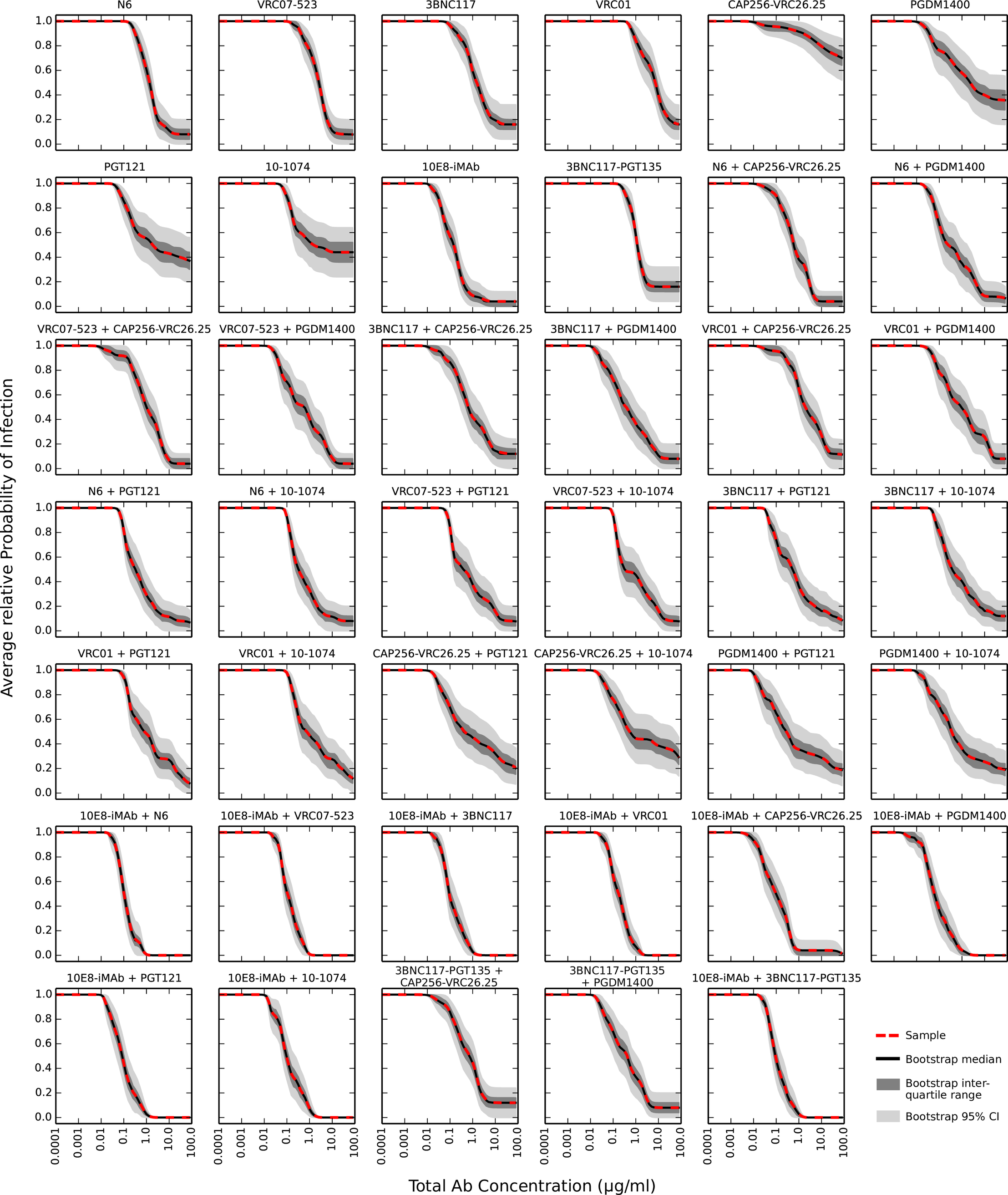

Supplement: S2 Fig — Each panel shows the average relative in vivo probability of infection as a function of concentration for individual Abs and combinations using the full subtype A pseudovirus panel data (red dashed curves) and using 1,000 bootstrap replicates (Methods). The bootstrap median curves are shown with black lines, the interquartile range (25–75 percentiles) at each concentration shown using dark grey shaded regions and the 95% confidence intervals shown using light grey shaded regions. (TIF) [file ppat.1006860.s003.tif]

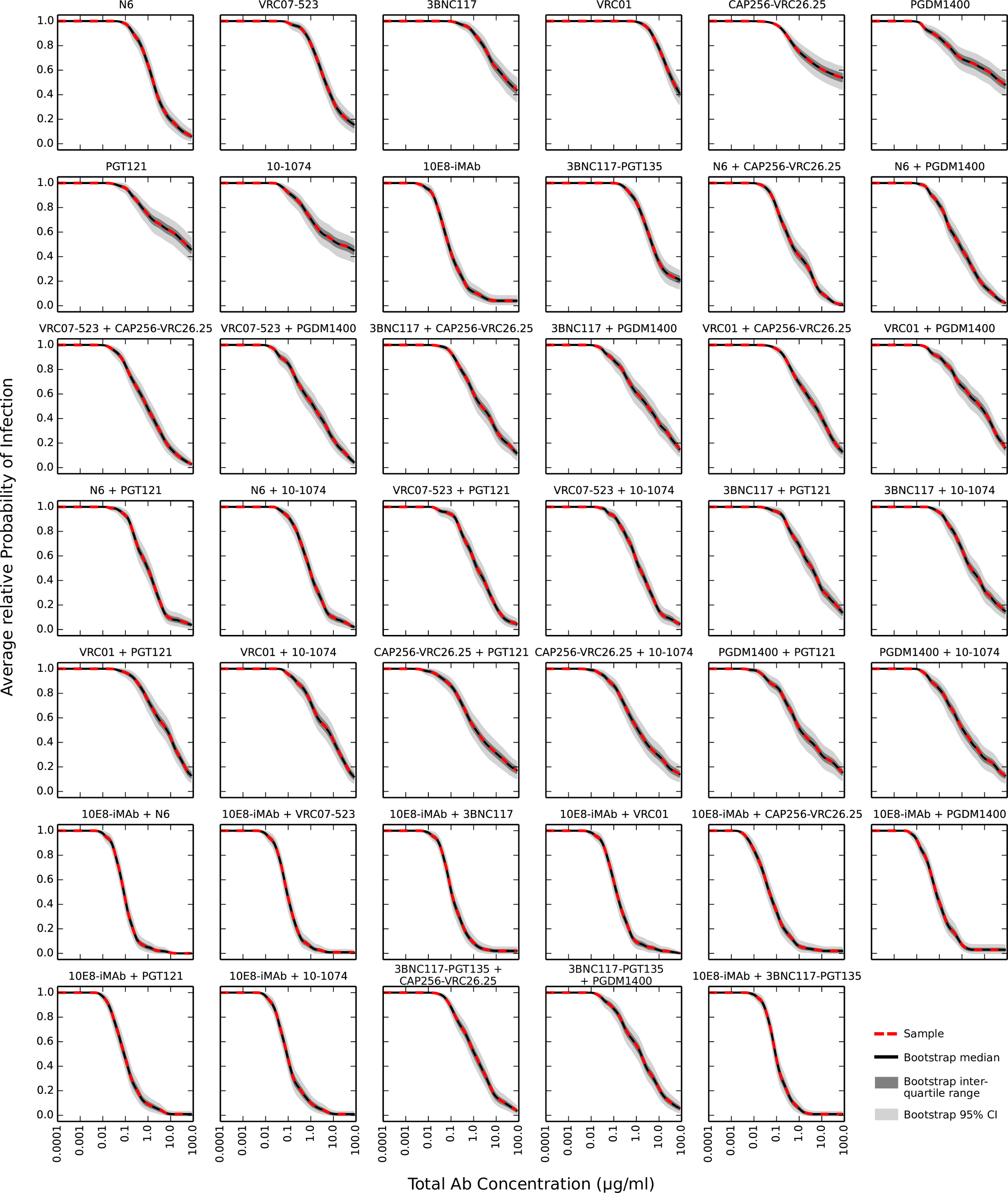

Supplement: S3 Fig — Same as S2 Fig, except using subtype C pseudovirus panel. (TIF) [file ppat.1006860.s004.tif]

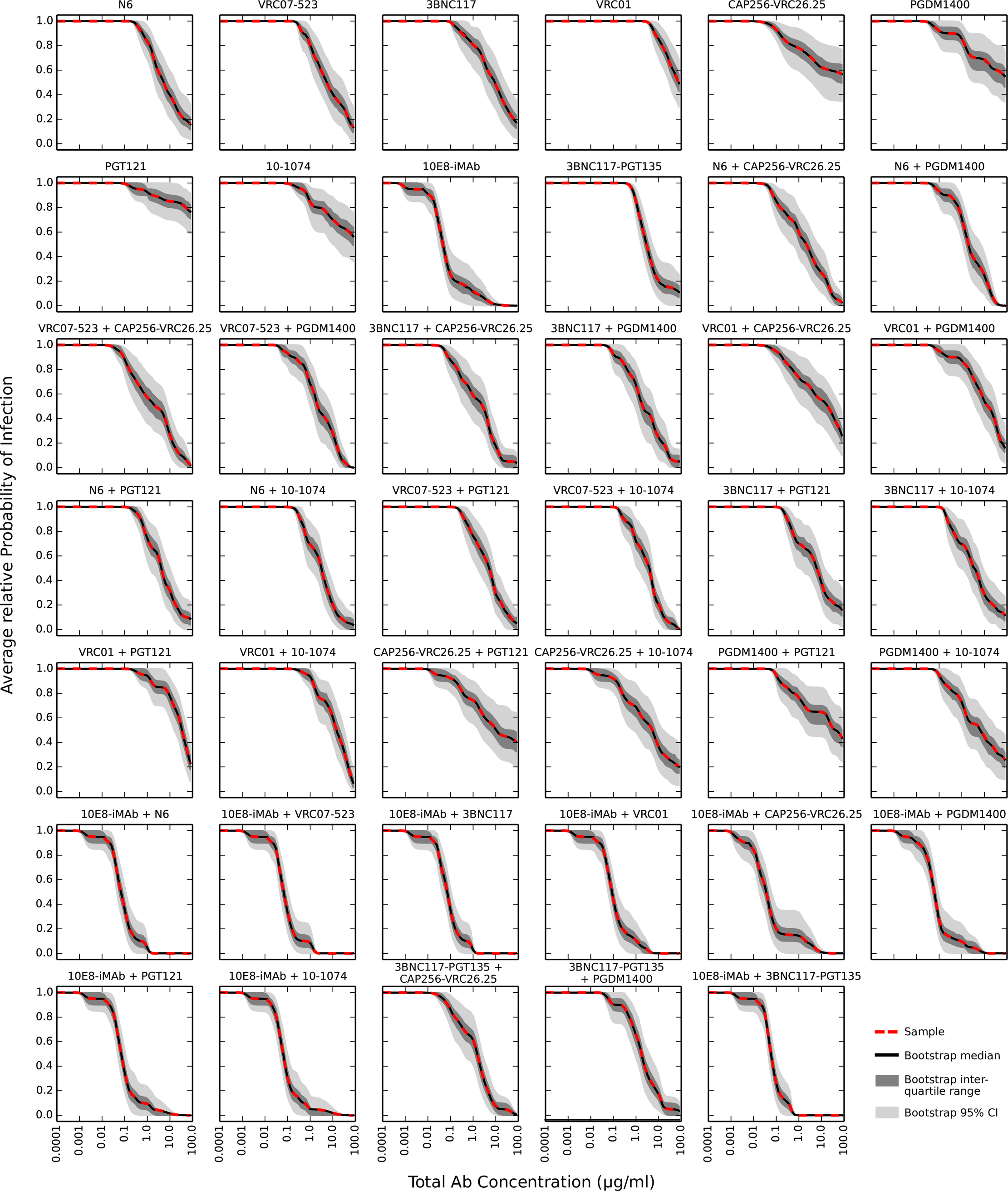

Supplement: S4 Fig — Same as S2 Fig, except using subtype D pseudovirus panel. (TIF) [file ppat.1006860.s005.tif]
